# 

**Published:** 1895-05

**Authors:** 


### CONTENTS.

COMMUNICATIONS.

The Origin of Pathological Tendencies.......... 209
The Present Needs in Dentistry.......... 214

MONTHLY DIGEST.

Operative Dentistry.......... 237

SELECTIONS.

The Lay Press and the Medical Profession.......... 236
Food for Thought.......... 252

NOTICES.

Tri-State Meeting.......... 253
Illinois State Dental Society.......... 253
The Horace Wells Permanent Memorial.......... 256
Bibliographical.......... 257

EDITORIAL.

Mississippi Valley Dental Association.......... 257
Personal.—Mississippi Valley Dental Society.......... 259
On to Baltimore.—By What Way?.......... 260

